# Enabling population protein dynamics through Bayesian modeling

**DOI:** 10.1093/bioinformatics/btae484

**Published:** 2024-07-30

**Authors:** Sylvain Lehmann, Jérôme Vialaret, Audrey Gabelle, Luc Bauchet, Jean-Philippe Villemin, Christophe Hirtz, Jacques Colinge

**Affiliations:** Université de Montpellier, Montpellier, 34000, France; LBPC-PPC CHU Montpellier, INM INSERM, Montpellier, 34000, France; LBPC-PPC CHU Montpellier, INM INSERM, Montpellier, 34000, France; Université de Montpellier, Montpellier, 34000, France; CMRR CHU Montpellier, INM INSERM, Montpellier, 34000, France; Université de Montpellier, Montpellier, 34000, France; Department of Neurosurgery, CHU Montpellier, INM INSERM, Montpellier, 34000, France; Université de Montpellier, Montpellier, 34000, France; Institut régional du Cancer Montpellier (ICM), Montpellier, 34000, France; Institut de Recherche en Cancérologie de Montpellier (IRCM), Inserm, Montpellier U1194, 34000, France; Université de Montpellier, Montpellier, 34000, France; LBPC-PPC CHU Montpellier, INM INSERM, Montpellier, 34000, France; CMRR CHU Montpellier, INM INSERM, Montpellier, 34000, France; Université de Montpellier, Montpellier, 34000, France; Institut régional du Cancer Montpellier (ICM), Montpellier, 34000, France; Institut de Recherche en Cancérologie de Montpellier (IRCM), Inserm, Montpellier U1194, 34000, France

## Abstract

**Motivation:**

The knowledge of protein dynamics, or turnover, in patients provides invaluable information related to certain diseases, drug efficacy, or biological processes. A great corpus of experimental and computational methods has been developed, including by us, in the case of human patients followed *in vivo*. Moving one step further, we propose a novel modeling approach to capture population protein dynamics using Bayesian methods.

**Results:**

Using two datasets, we demonstrate that models inspired by population pharmacokinetics can accurately capture protein turnover within a cohort and account for inter-individual variability. Such models pave the way for comparative studies searching for altered dynamics or biomarkers in diseases.

**Availability and implementation:**

R code and preprocessed data are available from zenodo.org. Raw data are available from panoramaweb.org.

## 1 Introduction

There is great interest in learning about the dynamics of proteins (Doherty and Whitfield 2011), beyond the knowledge of protein abundance in various tissues (Meyer and Schilling 2017). Protein dynamics is commonly referred to as protein turnover. It is the net rate at which proteins are produced or imported in a tissue, and simultaneously degraded or cleared. It provides a complementary perspective to protein abundance and it is relevant in a number of applications of clinical proteomics. For example, in various pathologies, abnormal turnover has been observed for specific proteins such as amyloid-β (Aβ), Tau, or sTREM2 in Alzheimer disease (AD) (Mawuenyega *et al.* 2010, Suárez-Calvet *et al.* 2016, Sato *et al.* 2018), retinol-binding protein 4 (RBP4) in diabetes (Jourdan *et al.* 2009), or tissue remodeling during early-stage human heart failure (Lam *et al.* 2014). Besides clinical applications, protein turnover may link to fundamental biological processes such as heart morphogenesis (Konzer *et al.* 2013). Turnover data are typically acquired by mass spectrometry (MS) after introducing an isotopic tracer to label the newly synthesized proteins (Bateman *et al.* 2006, Jaleel *et al.* 2006, Claydon *et al.* 2012, Doherty *et al.* 2012, Guan *et al.* 2012, Rahman *et al.* 2016, Sadygov *et al.* 2018, Wilkinson 2018, Sadygov 2022). Relative isotope abundance (RIA) is the ratio of labeled to unlabeled protein abundances. The variation of RIA over time provides turnover information.

Different protocols can be used to introduce a tracer, e.g. *via* the diet, intravenous injections, or even the medium if we consider cells in culture or organoids. Our interest here is in human—or animal—*in vivo* studies, where biofluids represent the most convenient and ethically acceptable material for sequential measures. Following an initial publication of our labeling protocol (Lehmann *et al.* 2015), we developed a flexible and accurate 2-compartment mathematical model (Lehmann *et al.* 2019). Specifically, we showed that this general model was able to fit data obtained by stable isotope labeling kinetics (SILK) (Bateman *et al.* 2006) accurately and it compared favorably with preexisting models achieving the same accuracy with fewer parameters (Lehmann *et al.* 2019). SILK is a pulse-chase protocol in which ^13^C_6_-Leu is injected intravenously for 9 h, allowing new protein synthesis but also clearance to be observed by collecting sequential samples over time, 24 h for instance. The new work we present here is a follow-up that brings the modeling, and the extraction of kinetic parameters, to the population level. Namely, given a cohort or population of individuals that have undergone SILK, we want to learn the typical values, variability, and correlation of the protein dynamics parameters over the whole population. This type of mathematical model is common in population pharmacokinetics (Bauer *et al.* 2007), where drug availability and clearance in patients need to be characterized population-wide in order to adjust standard regimens. In the case of protein turnover, our perspective is to provide robust and comprehensive models of healthy homeostatic states compared to disease states. This knowledge could obviously lead to advances in biomarker discovery as well as diagnostic applications beyond pure research and protein classification.

Searching for model parameters with classical optimization methods such as iterations that minimize the differences between model predictions and experimental data requires initial values for the searched parameters. The optimal parameters found may depend on the initial values if there are multiple local minima. In a complex hierarchical model consisting of a population level and then individual level parameters, this is a problem. A common and efficient solution involves Bayesian modeling, where prior knowledge of typical parameter values replaces discrete initial values. That is, instead of choosing one or more initial values, a whole distribution of initial values is considered. The observed data are then combined with this prior knowledge to infer the model parameters as statistical distributions (posteriors). If the experimental data are sufficient, the influence of the prior distribution is marginal and the problem of choosing the right initial values is eliminated. In addition, knowing the model parameters as posterior distributions provides information about their variability.

Accordingly, we decided to approach the problem of population protein dynamics with Bayesian modeling. Since this problem is very similar to population pharmacokinetics, we developed a hierarchical Bayesian model inspired by common practice in the field. Parameters were fitted using Markov-chain Monte-Carlo (MCMC) sampling. The new model is illustrated using an unpublished cohort of seven individuals whose blood plasma samples were analyzed by targeted MS, i.e. multiple reaction monitoring (MRM). To contrast this first, which showed moderate inter-individual variability, we used a second cohort of four individual cerebrospinal fluid (CSF) samples. The latter were not fully comparable and thus provided much more heterogeneous inter-individual data, allowing us to test the robustness of our population model.

## 2 Materials and methods

### 2.1 Human samples

Samples were generated following the clinical protocol “In Vivo Alzheimer Proteomics (PROMARA)” (ClinicalTrials Identifier: NCT02263235), which was authorized by the French ethical committee CPP Sud-Méditerranée IV (#2011–003926-28) and by the ANSM agency (#121457A-11). Enrolled patients (group a) were hospitalized in neurosurgery unit due to subarachnoid hemorrhage and received a temporary ventricular derivation of the CSF. The experiment protein turnover started 8–19 days after initial, medical ventricular drainage and normalization of CSF clinical chemistry analysis [normal CSF protein content lies in the 0.2–0.4 g/l range (Roche *et al.* 2008)]. Additional patients (group b) were hospitalized in neurology in relation with cognitive impairment etiologic investigation. Patient data are reported in Supplementary Table S1. CSF and blood plasma were collected at multiple time points after injection of the tracer for roughly 24 (CSF) or up to 36 (plasma) h. We applied the ethically approved (see above) original SILK 13C6-Leu infusion protocol (Bateman *et al.* 2006). Briefly, 13C6-Leu prepared per the European Pharmacopeia was intravenously administered. After a 10 min initial bolus at 2 mg/kg, an 8h50 infusion at 2 mg kg/h was performed. Ventricular CSF or plasma EDTA samples were collected starting at the beginning of the 13C6-leucine infusion, roughly every 3 h (3–6 ml). Samples were transported to the laboratory at 4°C, and centrifuged at 2000*g* for 10 min. CSF and plasma samples were aliquoted into 1.5-ml polypropylene tubes and stored at −80°C until further analysis.

In this study, we analyzed CSF samples from four patients of group a (Pat1a–Pat4a) and seven of group b (Pat7b–Pat13b). Patients were selected based on availability of CSF and plasma MS samples at multiple time points.

### 2.2 Sample analysis

Sample preparation was automated on AssayMap BRAVO (Agilent T., Santa Clara, United States) to reduce preanalytical variability. Briefly, 2 µl of plasma or 30 µl of CSF were used. Protein samples were reduced and alkylated, and digested with trypsin prior to LC–MS analysis.

**Figure 1.**
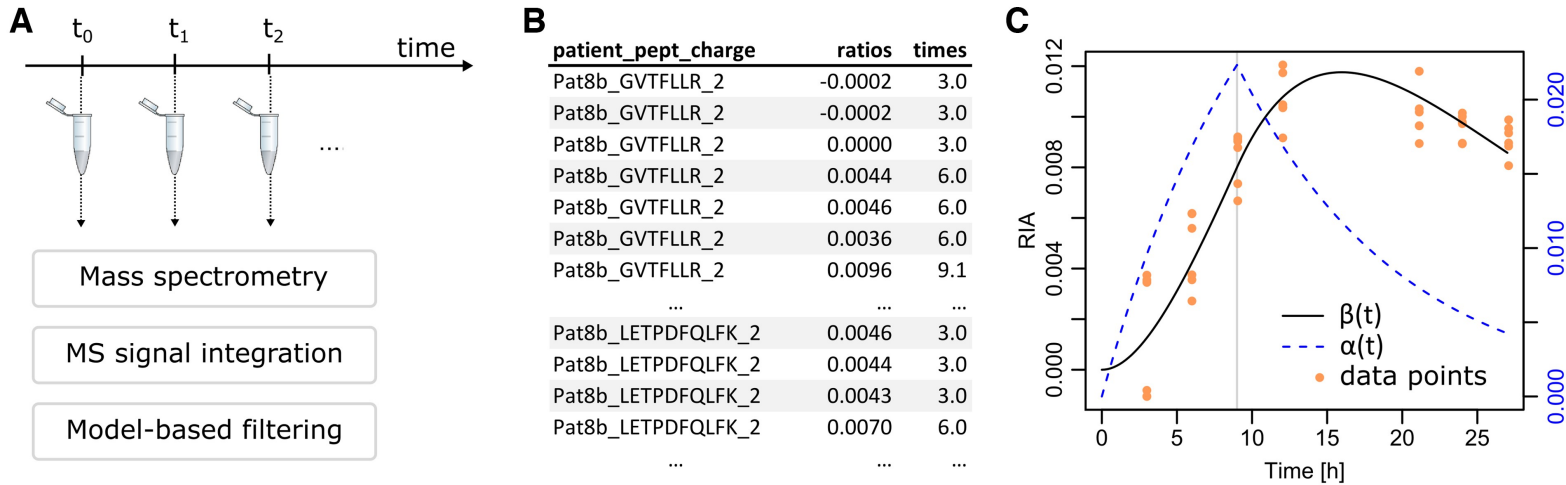
Simplified workflow. (A) Samples were collected at multiple time points and analysed by MS. Integration of all the spectra found for a given peptide at a given time point was performed with Skyline. Filtering of peptides for which sufficient and interpretable signals were available was performed employing a mathematical model of protein turnover. (B) Representative data for a given protein (A1BG), for which two different peptides were followed in MRM and both measured with double charges. Corresponding acquisition times and RIA (ratios) were available. (C) A1BG ratios (orange dots) along with the fitted mathematical model. The vertical line at 9 h indicates the end of tracer injection.

The MRM protocol was reported in previous publications (Percy *et al.* 2013, Hirtz *et al.* 2018, Lehmann *et al.* 2019); we hence only summarize the main steps here. Proteins were selected for their relevance to neurodegenerative diseases and clear detection in previous, proteome-wide experiments in plasma and CSF by our laboratory. The reporter peptides were selected for their high signal intensity in these previous experiments. Supplementary Tables S2 and S3 report the selected proteins and peptides for CSF and plasma samples. MRM was executed on the samples directly using a 1290 liquid chromatography (LC) system (Agilent Technologies) equipped with a reverse-phase column (RRHD Eclipse Plus C18) coupled with a QqQ MS instrument (6490, Agilent technologies). The MS instrument worked in dynamic MRM with a retention time window of 4.5 min and a maximum cycle time fixed at 700 ms. All the analyses were performed in duplicates. A minimum of one peptide per protein and three transitions by peptide were required. Skyline 4.1 was used to process raw MS data. Figure 1A presents an overview of the LC and MS pipeline.

Although they were not the object of this work, we also used two samples (CSF and plasma from PatA1) that were subjected to the proteome-wide (non MRM) protocol as describe in our previous publication (Lehmann *et al.* 2019). The CSF sample was discussed in this latter publication, whereas the blood sample remains unpublished. The data generated were subjected to the data analysis workflow described below. We obtained reliable turnover data for roughly 200 proteins in each sample (see Supplementary Tables S4 and S5), whose parameters were only used to learn the typical range of parameter values for mathematical modeling (see below) from diverse proteins.

### 2.3 MS data processing and existing individual mathematical model

Integrated MS spectra by Skyline were used as input to fit protein dynamics models. Typical input data are shown in Fig. 1B. The processing of these data has been described in detail previously (Lehmann *et al.* 2019), so we provide only a summary. For a given peptide and a given time, the observed RIA (Fig. 1B) is defined by the ratio of the heavy Leu signal $P_{H}$ (observed at a shifted mass of +6 Da *per* Leu) and the total signal $P_{L}+P_{H}$, $P_{L}$ the signal at the nominal mass. The injection of the tracer is modeled by a function f(t), with $f\left(t\right)=1$ for t≤9 and $f\left(t\right)=0$ for t>9. We denote the curve of RIA over time by β(t). Our two-compartment model is based on a notion of the rate of tracer biological availability of the tracer (first compartment) denoted α(t), which is involved in protein synthesis (second compartment). To parallel pharmacokinetic two-compartment models, α(t) relates to the ratio of initial drug concentration divided by the volume of distribution. Note that in the case of protein turnover, only ratios are modeled and therefore α and β are dimensionless. They are linked by the following ordinary differential equations (ODEs):

(1) $$\left\{\begin{array}{lll} \;\frac{d\alpha }{dt} & = & \left(\lambda f\left(t\right)-\alpha \right)k_{c},\\ \;\frac{d\beta }{dt} & = & \left(\alpha -\beta \right)k_{c}, \end{array}\right.$$

with $\alpha \left(0\right)=0=\beta (0)$. Figure 1C illustrates a typical dataset with the α and β curves. It is important to note that λ, which relates to tracer availability for protein synthesis, essentially acts as a scale parameter. The clearance/degradation rate $k_{c}$ acts primarily as a shape parameter that conditions the protein half-life. Due to the generally large ratio of intensities between $P_{L}$ and $P_{H}$, we have shown that noise causes an almost uniform vertical shift of the observed RIA values (Lehmann *et al.* 2019, Giroux *et al.* 2024). Therefore, we proposed an algorithm that includes the computation of an optimal shift along with the parameters λ and $k_{c}$ to adjust β(t) to the data. Summed squared errors with respect to observed RIAs were weighted proportionally to $\sqrt{P_{H}}$ (RIAs with stronger $P_{H}$ signals were more accurate). Weighted summed squared errors were minimized by a quasi-Newton iteration (function optim in R with method BFGS) to adjust the parameters. A quasi-Newton iteration is an optimization of the classical Newton iteration where the Jacobian matrix of the function to be minimized does not need to be recomputed at each step. Equation (1) was numerically integrated using the RADAU5 method (Hairer and Wanner 1996). RADAU5 is an implicit fifth order Runge–Kutta method adapted to stiff problems that includes a fourth order dense output (estimation at any time point). Although the dynamical systems corresponding to good quality data presented in all figures of this article are not stiff, low signals, or erroneous data can lead to stiff systems (Lehmann *et al.* 2019). Since the differential model is also used to filter the data, we practically had to deal with stiff cases and RADAU5 allowed us to do it quickly.

To achieve robust results in the presence of noisy RIAs, parameter fitting was iterative with a first application of the above to call outlier RIAs. RIAs were considered outliers provided they were located at a distance greater than half the difference between the minimum and maximum values of the first fitted β(t) model. A second application of the quasi-Newton method was then performed without the outliers. In addition, RIAs at time 0 were always considered outliers, as no tracer incorporation had yet occurred. Our original data processing pipeline ended with the application of a bootstrap to estimate confidence intervals. Here, we used the R library boot to perform a nonparametric balanced bootstrap (100 times), whereas the original publication used a parametric Gaussian bootstrap. Hereafter, we refer to the parameter and CI95 estimates obtained by this procedure as QNB for quasi-Newton-bootstrap.

## 3 Results and discussion

### 3.1 Initial Bayesian models

We began the construction of a Bayesian population model by first building a single individual Bayesian model equivalent to our QNB original procedure presented above. The posterior distributions of the parameters were estimated by Type II Maximum Likelihood. Assuming that $\text{RI}A_{i}$ to be the ith observation and $\beta_{i}$ the corresponding model value, we naturally have

$$\text{RI}A_{i}\;∼\;N\left(\beta_{i}-s,\sigma^{2}/w_{i}\right),$$

with i∈{1;…;n} and n the number of RIAs, $\beta_{i}=\beta (t_{i})$, $t_{i}$ the time at which $\text{RI}A_{i}$ was observed (due to replicates, several $t_{i}$ can be identical with different indices i), $w_{i}$ the weight proportional to $\sqrt{P_{H}}$ for observation i, and s the vertical shift to acknowledge the noise in the ratios (see Supplementary Materials and Methods). $N(a,b^{2})$ denotes a normal distribution with mean a and variance $b^{2}$.

To obtain a complete, hierarchical Bayesian model, we introduce prior distributions on the model parameters as well as the mean and variance of the error normal distribution. Note that following common practice in the field, the model parameters were log-transformed to use a normal prior. The resulting Bayesian formulation is the following:

(2) $$\begin{array}{lll} \frac{d\alpha }{dt} & = & \left(\lambda f\left(t\right)-\alpha \right)k_{c},\\ \frac{d\beta }{dt} & = & \left(\alpha -\beta \right)k_{c},\\ \ln\left(\lambda \right) & ∼ & N\left(\mu_{l\lambda },\sigma_{l\lambda }^{2}\right),\\ \ln\left(k_{c}\right) & ∼ & N\left(\mu_{lk_{c}},\sigma_{lk_{c}}^{2}\right),\\ s & ∼ & N\left(\mu_{s},\sigma_{s}^{2}\right),\\ \text{RI}A_{i} & ∼ & N\left(\beta_{i}-s,\sigma^{2}/w_{i}\right),\\ \sigma^{-2} & ∼ & \gamma \left(0.001,\;0.001\right). \end{array}$$

We denote by $\gamma \left(a,b\right)$ a Gamma distribution with shape a and rate b. The prior parameters for s, $\ln\left(k_{c}\right)$, and ln⁡(λ) were learned from a large number of models (roughly 200 distinct proteins) fitted with the QNB algorithm (see Supplementary Materials and Methods and Supplementary Tables S4 and S5). In the case of s, we set $\mu_{s}=0$, and $\sigma_{s}^{2}$ at $10^{-3}$ for the plasma samples and 1/500 for the slightly noisier CSF data. The gamma prior for $\sigma^{-2}$, i.e. the precision, is a commonly used vague (non-informative) prior.

**Figure 2.**
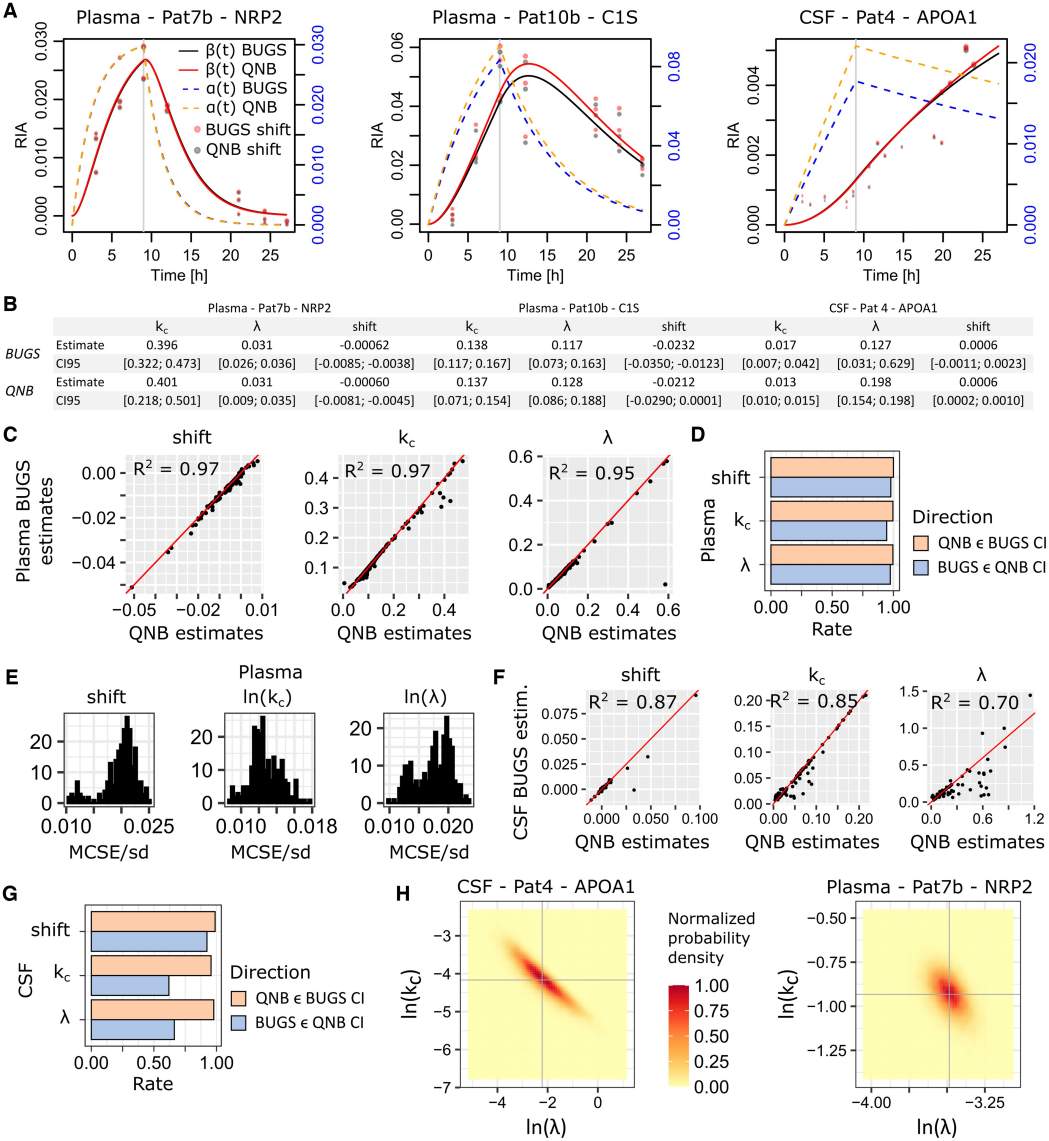
Initial Bayesian models of single proteins in single patients. (A) Three representative examples comparing the BUGS fitted models with the original QNB solutions. (B) Comparison of the BUGS versus QNB fitted parameters shift, $k_{c}$ and λ. Spearman squared correlation is denoted $R^{2}$. (C) Global comparison of BUGS versus QNB-estimated parameters over all the plasma proteins and patients. (D) Rate of inclusion of the QNB parameter estimates in the BUGS 95% credible intervals, and conversely. (E) Autocorrelation-corrected MCSE/estimate standard deviation distributions. (F) BUGS versus QNB parameter estimate correlation over all the CSF proteins and patients. (G) Inclusion rates of QNB estimates in BUGS 95% credible intervals and *vice versa*. (H) MCMC sampled probability density over the $\left(\lambda ,k_{c}\right)$-space. Marginal means are featured by the gray crosses.

We implemented MCMC sampling for σ, s, $\ln\left(k_{c}\right)$, and ln⁡(λ) in the above model using two approaches, both implemented in R. First, we defined a function proportional to the log probability density of (2) and used the R libraries mcmc (function metrop, Metropolis algorithm) and adaptMCMC [function MCMC, robust adaptive Metropolis (Vihola 2012)]. Alternatively, we used BUGS to define the model and OpenBUGS (Lunn *et al.* 2000) through its R interface R2OpenBUGS. Some particular β(t) shapes such as apolipoprotein A1 (APOA1) in CSF illustrated in Fig. 2A were more difficult to fit, and adaptMCMC and mcmc failed to find correct parameters (Supplementary Fig. S1). OpenBUGS managed to handle these more difficult data efficiently. In the majority of cases, typically illustrated by neuropilin-2 (NRP2) and complex component 1s (C1S) in Fig. 2A, the three libraries produced nearly identical parameter estimates (Supplementary Fig. S1). Thus, the distribution-aware OpenBUGS Gibbs sampler was more effective for our application. We decided to use only OpenBUGS. The BUGS code and the R function for adaptMCMC and mcmc are provided in Supplementary Information. We found that 100 000 iterations including 50 000 burn-in were sufficient for OpenBUGS to converge safely. We systematically used two Markov chains, and a convergence diagnostic was obtained by comparing within- and between-chain variability (Brooks and Gelman 1998). Each chain was initialized with random λ, and $k_{c}$ values drawn from their respective prior distributions. The shift s was initialized at 0 and $\sigma^{2}$ at 0.1.

Outliers identified by the QNB algorithm were removed from the data given to OpenBUGS (same for mcmc and adaptMCMC). The latter was indeed sensitive to some extreme outliers (data not shown). Theoretically, it would be possible to replace the normal distribution for the errors on $\text{RI}A_{i}$ in Eq. (2) with a heavy-tailed distribution such as Student with 4 degrees of freedom to address outliers. However, our goal here was not to replace the satisfactory QNB algorithm, but to build a population model on top of it. We thus exploited QNB outlier calling.

Figure 2A shows that the Bayesian solution was usually close to QNB, with NRP2 and C1S being representative of the majority of cases. The inferred parameters for the two algorithms are reported in Fig. 2B with estimated 95% confidence intervals (QNB) and 95% credible intervals (BUGS). Indeed, when comparing the parameter values for all the plasma proteins in patients Pat7b–Pat13b (37 different proteins, 236 individual proteins in total, Supplementary Table S3), we found an excellent correlation (Fig. 2C). We also found a high agreement between the 95% confidence and credible intervals (Fig. 2D). As a general rule of thumb, the number of iterations in an MCMC estimation should be such that the MCMC standard error (MCSE) divided by the standard deviation (SD) of the sampled parameter remains below 5%. As shown in Fig. 2E, this was achieved for the three estimated parameters using a conservative value for the MCSE that was corrected for autocorrelation (OpenBUGS “Time-series SE” estimates). This validated the choice of the number of iterations. Convergence according to Brooks and Gelman criterion is shown in Supplementary Fig. S2.

Considering CSF data (Pat1–Pat4, 26 different proteins, 92 individual proteins in total, Supplementary Table S2), we made similar observations (Fig. 2F), but with a lower correlation. This is explained by the fact that in the CSF data, more difficult β(t) shapes similar to APOA1 in Fig. 2A roughly represented half the data. Credible intervals estimated by OpenBUGS were reliable and almost always included QNB estimates, while the converse was more in the 70% range, but for the shift that was highly compatible with QNB estimates. MCSE/SD values remained below 5% and convergence was achieved, see Supplementary Figs S3 and S4.

To better understand the CSF results, we first observed that in the typical example of APOA1 (Fig. 2A), the β(t) curve was correctly fitted to the experimental RIAs by both algorithms. However, the QNB and BUGS α(t) curves were different. This indicated a stronger correlation between the model parameters λ and $k_{c}$ that manifested by the ability to compensate variation of one by the value of the other. Indeed, comparing the probability density estimated by MCMC sampling over the $\left(\lambda ,k_{c}\right)$-space, we found a larger and narrower region for CSF Pat4 APOA1, but a more confined and rounder region for plasma Pat7b NRP2 (Fig. 2H). The latter configuration is usually easier to explore and leads to faster convergence, whereas the former is often associated with slower convergence due to inefficient exploration of the parameter space. This was also reflected in the BUGS credible interval sizes in Fig. 2B. The Bayesian approach was better at estimating realistic 95% credible intervals, almost always including QNB estimates. This was not the case for the QNB-estimated 95% confidence intervals, which tended to be too narrow including no more than ∼70% of the BUGS estimates. Lastly, it is important to remember that in Eq. (1), the most relevant, shape or half-life related parameter is $k_{c}$ for which a rather high $R^{2}=0.85$ (Spearman) was obtained. All the model parameter estimates for all the CSF and plasma models are available as OpenBUGS output in Supplementary Data.

### 3.2 Population hierarchical Bayesian model

**Figure 3.**
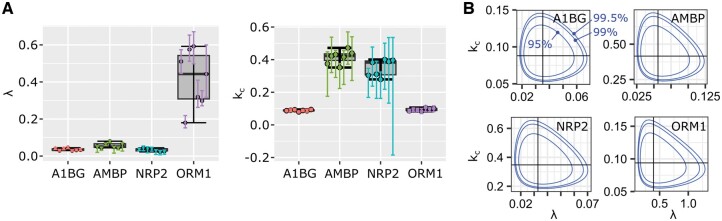
Four example proteins in plasma data. (A) Variability of individual parameters for the seven patients. Dots represent QNB estimates, the vertical color bars their respective CI95s, and a boxplot computed form the individual estimates was added to the background to suggest population dispersion. (B) Areas covered in the $\left(\lambda ,k_{c}\right)$ parameter space by the true Bayesian population models. Concentric boundaries indicate the space occupied by 95% (inside the innermost boundary), 99% and 99.5% of the population as estimated from the available cohort.

To develop a population model, we start with plasma data, typical examples are featured in Fig. 3A (the 27 plasma proteins for which we had data for every patient are depicted in Supplementary Fig. S5).

The principle of population Bayesian modeling consists in adding a population level to the model in Eq. (2). This additional level should capture the typical values of the parameters as well as potential correlations between them (Fig. 2H). If we define the parameter vector

$$\theta =\left(\begin{array}{l} \ln\left(\lambda \right)\\ \ln(k_{c}) \end{array}\right),$$

then this is achieved by a 2D normal distribution

$$\theta \;∼\;N_{2}\left(\mu_{\theta },\Omega \right),$$

with mean $\mu_{\theta }$ and variance Ω. Hyper-priors are introduced for these two quantities

$$\begin{array}{lll} \mu_{\theta } & ∼ & N_{2}\left(\mu ,\Sigma \right),\\ \Omega^{-1} & ∼ & {\text{Wi}}_{2}\left(R,2\right), \end{array}$$

where the vector $\mu \in R^{2}$ components are respectively set to the means of ln⁡(λ) and $\ln\left(k_{c}\right)$. Those means were obtained from a large set of previous observations as we did for the individual models above (Supplementary Table S4). For Σ, we employed a commonly used vague hyper-prior with

$$\Sigma^{-1}=\left(\begin{array}{ll} 10^{-4} & 0\\ 0 & 10^{-4} \end{array}\right).$$

${\text{Wi}}_{2}(R,2)$ denotes a 2D Wishart distribution that generalizes the Gamma distribution to multidimensional variates (compare $\Omega^{-1}$ above with $\sigma^{-2}$ in Eq. (2)). A widely used vague hyper-prior is obtained with

$$R=\left(\begin{array}{ll} 0.175 & 0\\ 0 & 0.175 \end{array}\right).$$

Lastly, writing ${\text{RIA}}_{ki}$ the ith observed RIA for patient k, the residuals likelihood is defined by

(3) $$\begin{array}{lll} \frac{d\alpha_{k}}{dt} & = & \left({\;\text{exp}(\theta }_{1k})\;f\left(t\right)-\alpha_{k}\right)\;\;\text{exp}\;\left(\theta_{2k}\right),\\ \frac{d\beta_{k}}{dt} & = & \left(\alpha_{k}-\beta_{k}\right)\;\;\text{exp}\;\left(\theta_{2k}\right),\\ \theta_{k} & ∼ & N_{2}\left(\mu_{\theta },\Omega \right),\\ \mu_{\theta } & ∼ & N_{2}\left(\mu ,\Sigma \right),\\ \Omega^{-1} & ∼ & {\text{Wi}}_{2}\left(R,2\right),\\ s_{k} & ∼ & N\left(\mu_{s},\sigma_{s}^{2}\right),\\ \text{RI}A_{ki} & ∼ & N\left(\beta_{ki}-s_{k},\sigma^{2}/w_{ki}\right),\\ \sigma^{-2} & ∼ & \gamma \left(0.001,\;0.001\right), \end{array}$$

with (similar to the individual model) $\beta_{ki}$ the value of $\beta_{k}(t)$ at the time where ${\text{RIA}}_{ki}$ was observed, and the corresponding weight $w_{ki}$. Then, $\theta_{1k}$ is $\ln\left(\lambda_{k}\right)$ and $\theta_{2k}$ is $\ln(k_{ck})$, the logarithms of patient k turnover parameters.

As with the individual models, we ran the BUGS model (reported in Supplementary Information) for 100 000 iterations including 50 000 burn-ins, using two chains. The Markov chains were initialized with random $\theta_{k}$ drawn from their prior, $s_{k}=0$, and $\sigma^{2}=0.01$. Convergence was achieved for all the proteins except alpha-1-microglobulin/bikunin precursor (AMBP), beta-2-microglobulin (B2M), C1S, and NRP2. Running the BUGS models, same data and initial chains, with WinBUGS that offers real-time visual tracking, we found that θ components diverged in opposite directions. One parameter became infinitely large, and the other one became infinitely small to compensate. We could easily solve these four cases by starting the two chains with the parameter values $\theta_{k}$ set to the QNB estimates. Alternatively, an informative prior $N_{2}(\mu ,\Sigma )$ with μ and Σ learned from the seven QNB estimates available for each protein gave similar results (μ was set to the average, and diagonal elements of $\Sigma^{-1}$ to 1/variances). Applying these two alternative priors to the 23 proteins for which the vague prior worked, we found no real differences in the learned population models (Supplementary Fig. S6A and B). This indicates that the data were strong enough to eliminate any potential bias introduced by a specific prior. The four cases that failed with the vague prior (AMBP, B2M, C1S, and NRP2) shared the same fast turnover dynamics. The two alternative priors produced similar estimates for these four proteins (Supplementary Fig. S6C). Supplementary Figure S7 features seven proteins with individual dynamics and population dynamics represented by posterior predictive distributions to further illustrate the lack of bias introduced by the prior. Based on these results, we opted for the informative prior and obtained *bona fide* population parameters for the 27 plasma proteins. Control plots to justify the number of iterations and the convergence with this specific prior are reported in Supplementary Fig. S8. Figure 3B illustrates the population models for the example proteins in Fig. 3A. In particular, Fig. 3B shows the population probability density over the parameter space $\left(\lambda ,k_{c}\right)$. We note that the additional area of parameter space covered when moving from 95% to 99% or 99.5% of the population is very small indicating a robust estimate of turnover diversity for each protein.

**Figure 4.**
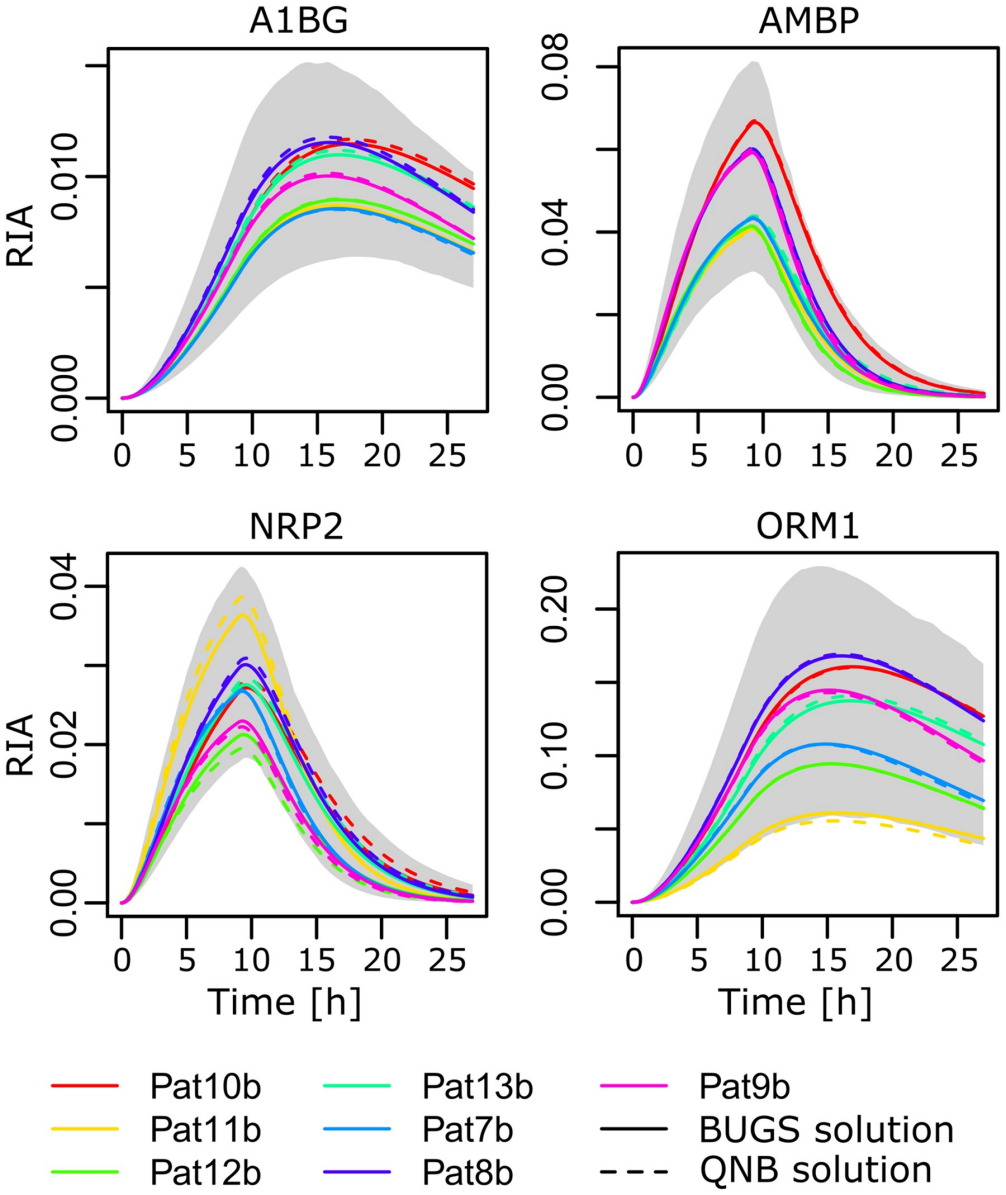
Population dynamics in plasma samples. Areas in the (time, RIA)-space that are covered by 95% of the population as estimated by our model based on the available cohort. The gray 95% area was obtained by generating 500 (*λ*, *k_c_*) pairs and computing the CI95 of all the 500 resulting *β*(*t*) curves at each time point. The individual curves for the BUGS model were obtained from the population model (3) using patient-specific *θ_k_* values. They were as accurate as the solutions found by individual BUGS model using Eq. (2) and illustrated in Fig. 2A.

Another advantage of a population model is that it allows us to estimate the range of protein dynamics that can be expected from 95% of the population, or any percentage of interest. This is done simply by simulating the predictive posterior distribution of the population model $N_{2}(\mu_{\theta },\Omega )$. In Fig. 4, four examples of protein dynamic ranges are featured along with the individual curves fitted from the same population model (Eq. (3)), but using $\theta_{k}$ values.

### 3.3 Population modeling of heterogeneous data

The CSF protein data showed a much higher inter-patient heterogeneity than the plasma dataset. This variability was due to the fact that the four CSF patients experienced subarachnoid hemorrhage, which introduced blood into the CSF. Part of their treatment included ventricular CSF drainage, which provided the opportunity to collect the samples. Although these samples were collected at a late stage of the therapy, when the CSF protein concentration was judged to have returned to a normal range (Supplementary Table S1), we cannot consider the CSF samples to be fully comparable. In this methodological work, where no biological conclusions are drawn, this provided an opportunity to confront our Bayesian approach with much more variable data.

**Figure 5.**
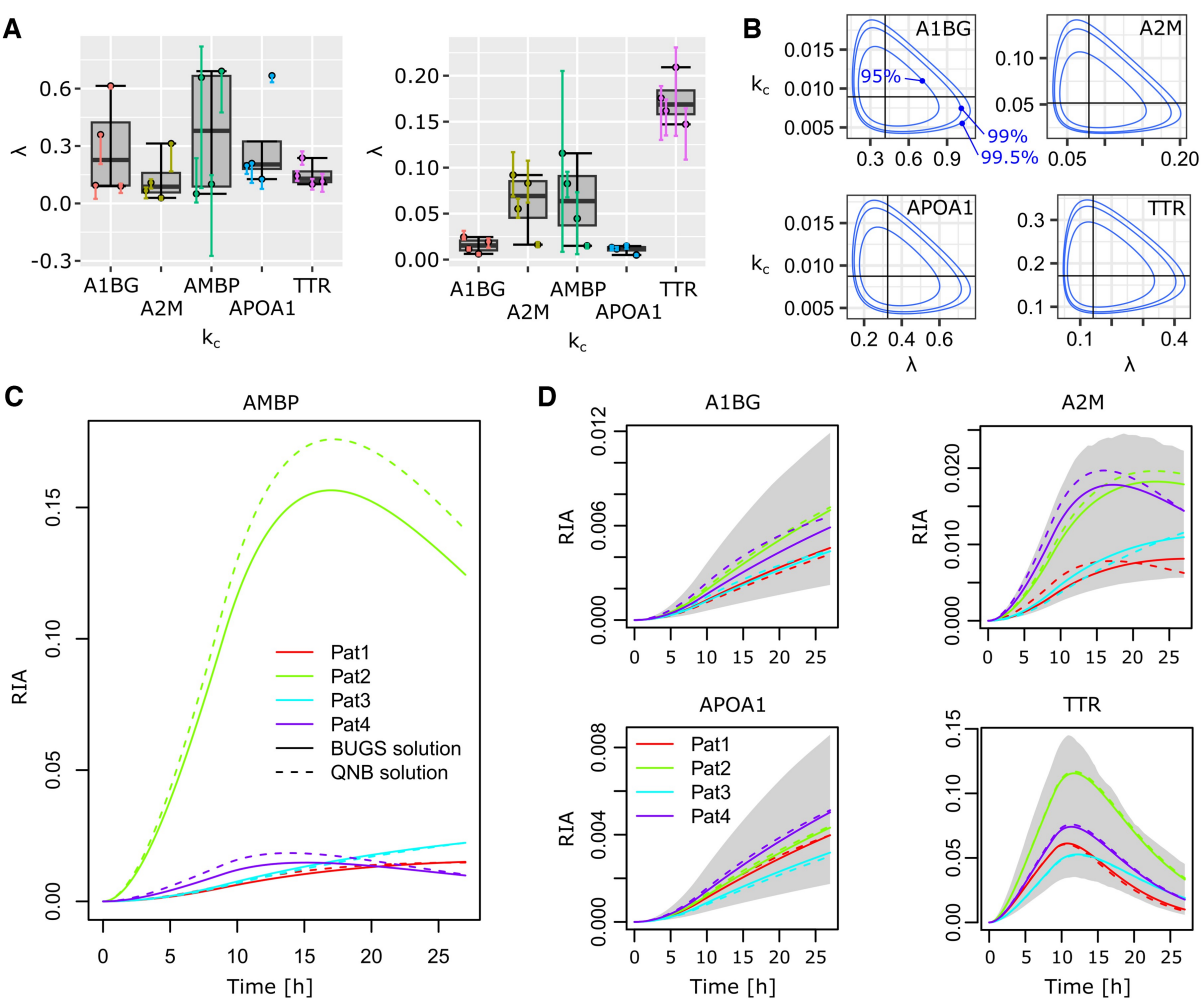
Modeling heterogeneous dynamics. (A) Individual parameter variability in the four CSF patients from group a. (B) Areas in the areas covered in the (*λ*, *k_c_*) parameter space by the true Bayesian population models. Concentric boundaries indicate the space occupied by 95% (inside the innermost boundary), 99% and 99.5% of the population as estimated from the available cohort. We could not build a population model for AMBP. (C) Extreme heterogeneity for AMBP individual dynamics with a very strong outlier. (D) Population dynamics, the gray area represents 95% of the population dynamics according to the model based on available data.

Of the 19 proteins for which we had data for all four patients, 12 could be modeled using a vague prior with random initial values for $\theta_{k}$ (θ prior parameters were trained on a set of roughly 200 different CSF proteins, Supplementary Table S5). The vague prior for $\theta_{k}$ with initial values from QNB estimates did not improve to the contrary of what we observed in plasma data. The $\theta_{k}$ informative prior trained on individual QNB estimates (see above) could nonetheless improve and resulted in 18/19 successful models. The AMBP model remained unestimated. Figure 5A illustrates different CSF cases covering all configurations of heterogeneity in signal intensity (λ) and turnover ($k_{c}$). See Supplementary Fig. S9 for all the 18 models and Supplementary Fig. S10 for information on sampling convergence. Figure 5B shows the population coverage of the parameter space. Figure 5C explains why CSF AMBP was so refractory to population modeling, and Fig. 5D illustrates the population dynamics. We note that our population model was successful in heterogeneous cases such as A2M or TTR (Fig. 5D).

## 4 Conclusion

In a previous publication, we introduced a mathematical model along with a data processing and filtering pipeline to analyze protein turnover data (Lehmann *et al.* 2019). This methodology, which we refer to as QNB in this article, enabled us to obtain turnover parameters for individual proteins in individual samples. Here, we have proposed an extension to integrate the population level, i.e. the variability within a cohort of individuals for each protein. Population-level modeling relied on a hierarchical Bayesian approach combined with MCMC sampling to infer model parameters. This is an approach used in population pharmacokinetic studies (Duffull *et al.* 2005, Bauer *et al.* 2007). Population modeling of protein turnover allowed us both to describe inter-individual variability in terms of the typical regions of the parameter space, and to derive accurate, individual-specific models capable of accurately inferring turnover parameters. We also showed that the choice of a specific prior had no real impact on the posterior distribution due to sufficient experimental data.

Two MRM datasets were exploited. One human blood plasma dataset exhibited limited inter-individual variability resulting in relatively easy modeling. Another human ventricular CSF dataset featured high inter-individual variability, which challenged our methodology. We were able to obtain accurate models for 18/19 proteins available for each patient, the last protein being pathological with one patient having massively different dynamics. For a larger cohort with high variability, one could imagine to identify subgroups of patients beforehand, or to include the notion of subgroup in the hierarchical Bayesian model, which is in principle well suited for such tasks. This possibility needs to be explored in future work with more data.

This study has established a new type of mathematical model for the protein turnover community, which we believe should greatly facilitate the description and comparison of natural and pathological protein turnover at the most relevant scale, the population.

## Supplementary Material

btae484_Supplementary_Data
