# Supplementary material for: Enabling population protein dynamics through Bayesian modeling
Source: Bioinformatics. 2024 Jul 30;40(8):btae484. doi: 10.1093/bioinformatics/btae484 (PMC11335370; doi:10.1093/bioinformatics/btae484)
Supplement: btae484_Supplementary_Data [file btae484_supplementary_data.zip › SI-SILK-2-version-5.docx]

Supplemental Information

**Enabling population protein dynamics through Bayesian modeling**

Sylvain Lehmann^1,2^, Jérôme Vialaret^2^, Audrey Gabelle^1,3^, Luc Bauchet^1,4^, Jean-Philippe Villemin^1,5,6^, Christophe Hirtz^1,2,3^, Jacques Colinge^1,5,6^

^1^Université de Montpellier, Montpellier, France

^2^LBPC-PPC CHU Montpellier, INM INSERM

^3^CMRR CHU Montpellier, INM INSERM

^4^Department of Neurosurgery, CHU Montpellier, INM INSERM

^5^Institut régional du Cancer Montpellier (ICM), Montpellier France

^6^Institut de Recherche en Cancérologie de Montpellier (IRCM), Inserm U1194, Montpellier, France

**SUPPLEMENTARY TABLES & FIGURES**

**Table S1.** Demographic information (gray area irrelevant for plasma samples).

| **Patient** | **Sex** | **Age** | **Diagnosis** | **Time after set up of ventricular drainage** | **CSF protein concentration (g/L)** | **Cell count per mm^3^** |
| --- | --- | --- | --- | --- | --- | --- |
| Pat1a | F | 67 | Subarachnoid hemorrhage, Fisher scale 4, left carotid aneurysm | 19 days | 0.34 | 40 |
| Pat2a | F | 65 | Subarachnoid hemorrhage, Fisher scale 4, anterior communicating artery aneurysm | 15 days | 0.15 | 10 |
| Pat3a | F | 49 | Subarachnoid hemorrhage, Fisher scale 3, cerebral right posterior artery aneurysm | 23 days | NA | NA |
| Pat4a | F | 68 | Subarachnoid hemorrhage, Fisher scale 3, cerebral anterior artery aneurysm | 8 days | 0.50 | 150 |
| Pat7b | M | 57 | Fronto-temporal dementia |  |  |  |
| Pat8b | F | 69 | Alzheimer’s disease |  |  |  |
| Pat9b | F | 49 | Mild Cognitive Impairment |  |  |  |
| Pat10b | F | 80 | Alzheimer’s disease |  |  |  |
| Pat11b | M | 77 | Mild Cognitive Impairment |  |  |  |
| Pat12b | F | 69 | Alzheimer’s disease |  |  |  |
| Pat13b | F | 61 | Alzheimer’s disease |  |  |  |

**Table S2.** CSF proteins and peptides measured in MRM. See Supplementary Data.

**Table S3.** Plasma proteins and peptides measured in MRM. See Supplementary Data.

**Table S4.** Proteome-wide, unbiased analysis of Pat1 198 CSF proteins, turnover parameters. See Supplementary Data.

**Table S5.** Proteome-wide, unbiased analysis of Pat1 182 plasma proteins, turnover parameters. See Supplementary Data.


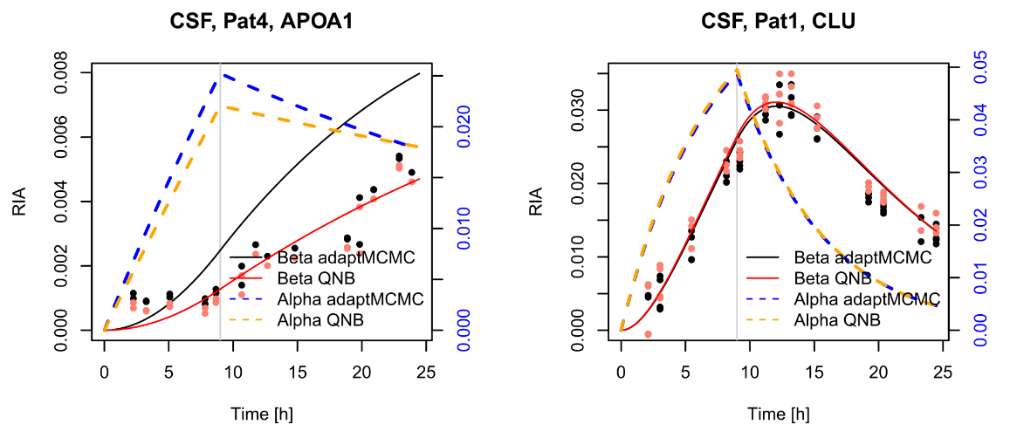


**Figure S1.** Failed and successful representative applications of adaptMCMC. (Left) An example where OpenBUGS managed to estimate model parameters leading to $\beta(t)$ going through the data points, while adaptMCMC inferred a similar shift but inadequate $\lambda$ and $k_{c}$. Data points are plotted in red and black according to the respective shifts of the two samplers. Here, 100 000 iterations with 50 000 burn-ins were used. To increase the number of iterations for adaptMCMC or to use its parallelized version with multiple chains did not improve, it always converged on this type of solution for data sets with this type of $\beta(t)$ geometry (data not shown). The same occurred with the library mcmc and its two samplers (data not shown). (Right) In the majority of cases, OpenBUGS and adaptMCMC or mcmc found very close and correct solutions as exemplified here with clusterin (CLU).


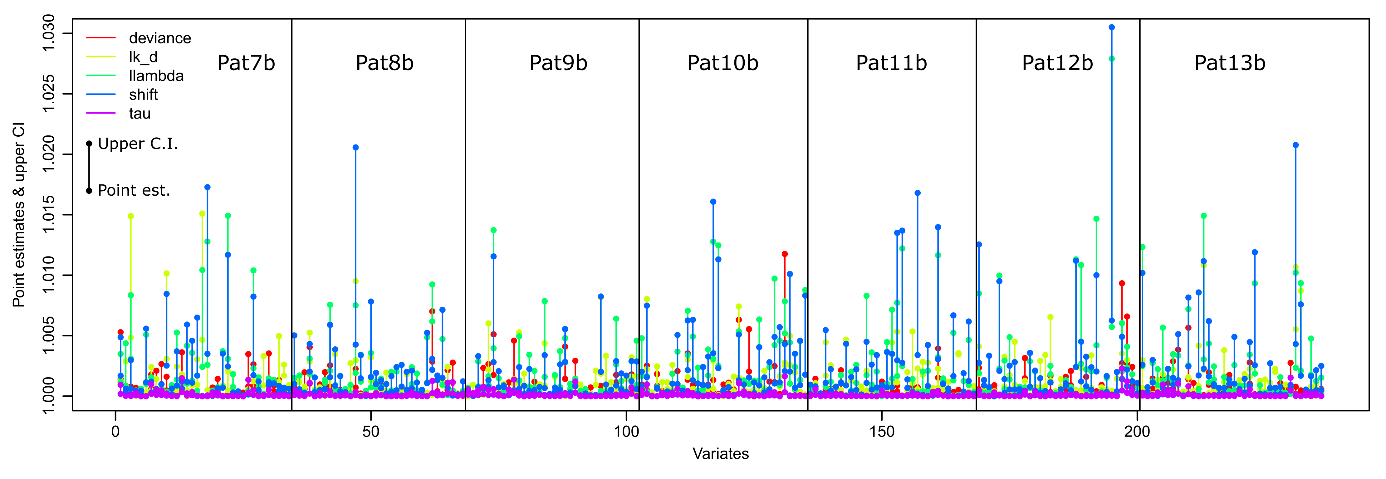


**Figure S2.** Convergence according to Brooks and Gelman criterion (1998) in plasma. Two Markov chains were run in parallel and the within- *versus* inter-chain variability ratio was computed. Point estimates along with the upper boundaries of 95% confidence intervals are plotted for all the model parameters and all the proteins available for all the patients. We see that ratios were below the recommended 1.05 threshold.


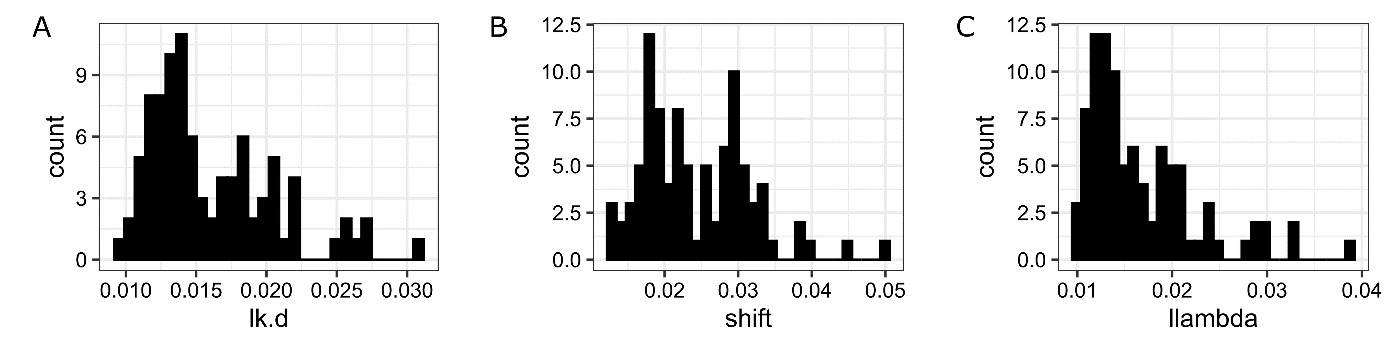


**Figure S3.** CSF individual models. Autocorrelation-corrected MCSE/estimate standard deviation distributions.


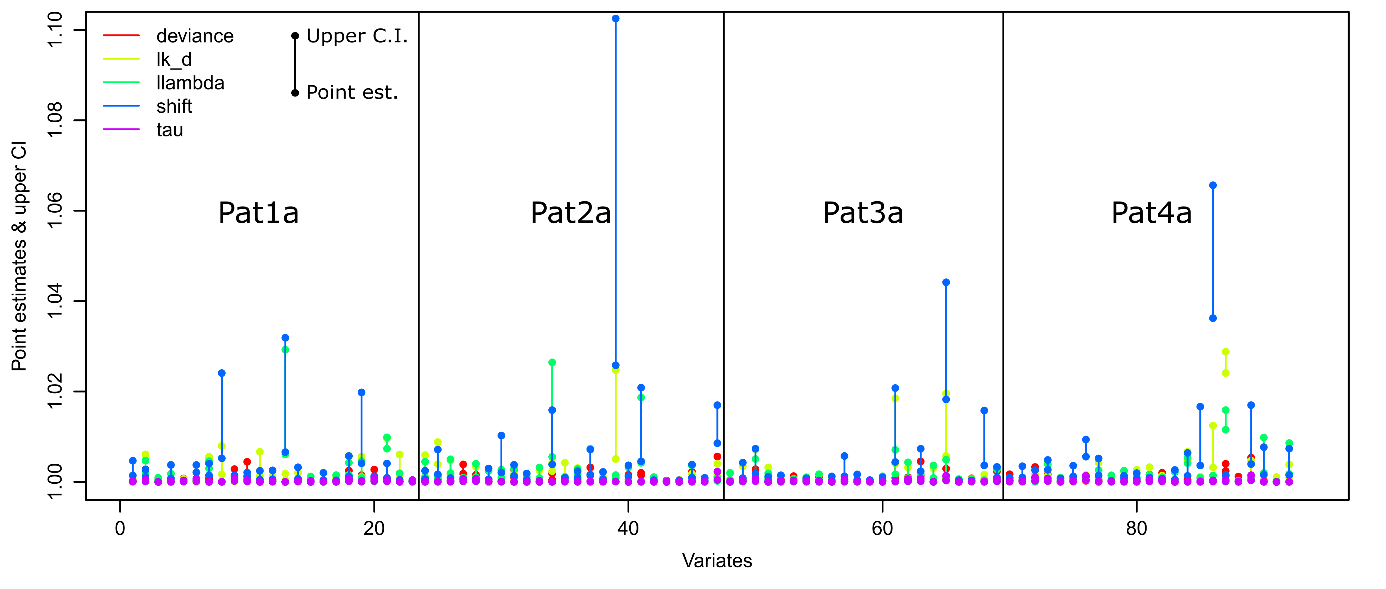
F**igure S4.** Convergence according to Brooks and Gelman criterion (1998) in CSF. Two Markov chains were run in parallel and the within- *versus* inter-chain variability ratio was computed. Point estimates along with the upper boundaries of 95% confidence intervals are plotted for all the model parameters and all the proteins available for all the patients. We see that all the ratio point estimates were below the recommended 1.05 threshold. In two cases (GC in Pat2a and Pat4a), the upper boundary of the CI95 was slightly above 1.05.


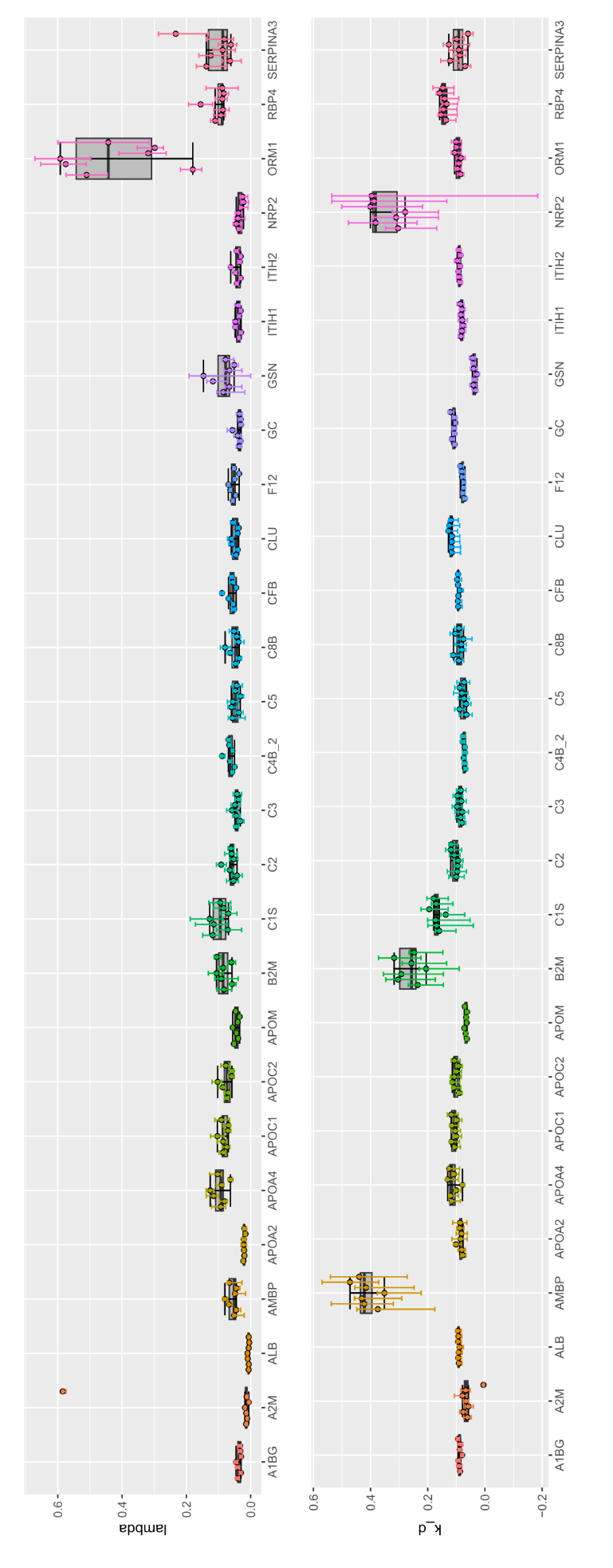


**Figure S5.** Blood plasma individual model parameter variability.


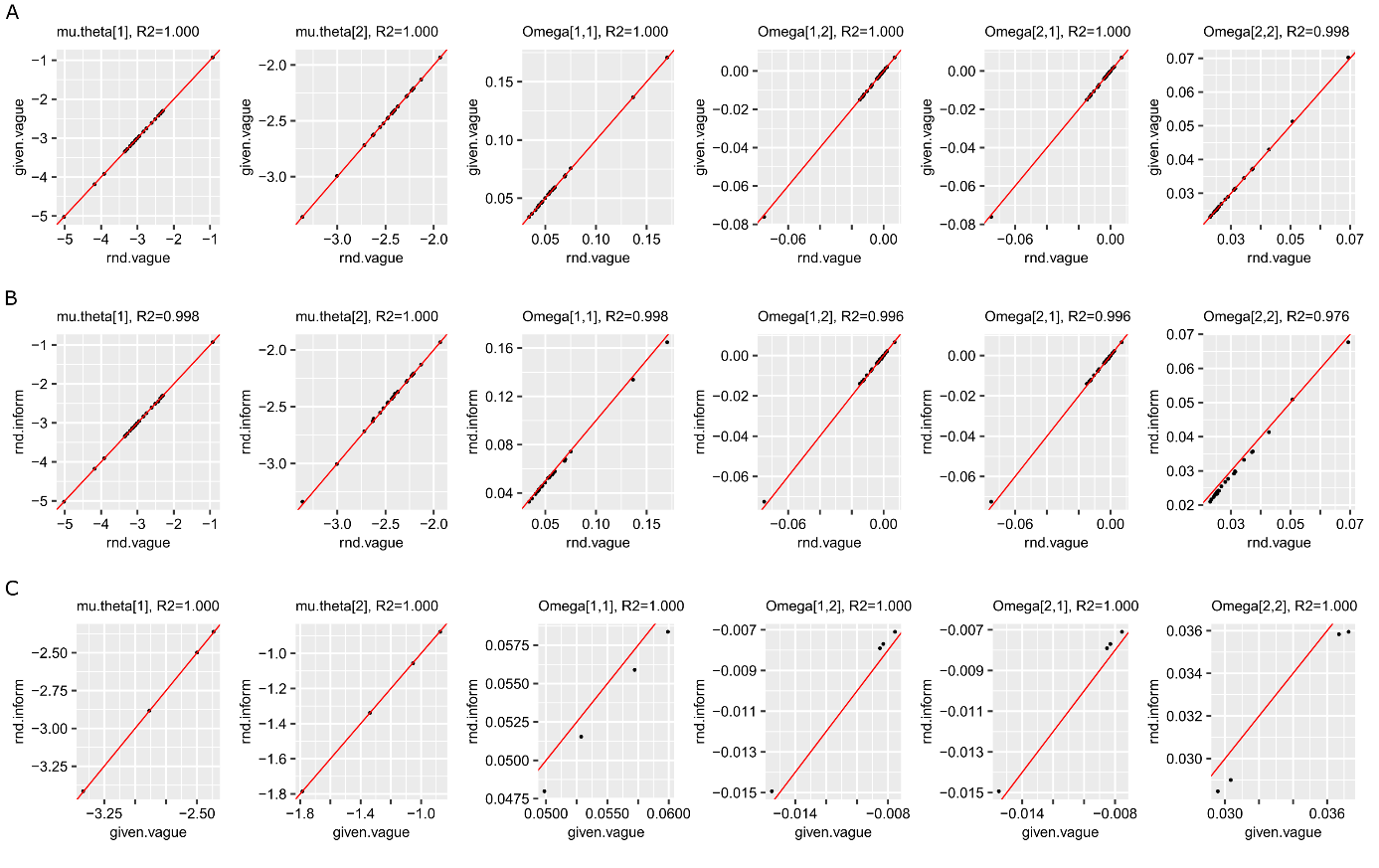


**Figure S6.** Prior specification. (A) Vague prior with random initial values (x-axis) *versus* vague prior with initial values estimated by the QNB algorithm (y-axis). Data from the 23 plasma proteins for which the vague prior with random initial values converged. (B) Vague prior with random initial values (x-axis) *versus* informative (hyper-)prior on $\mu_{\theta}$ with random initial values (y-axis). (C) Informative prior with random initial values (y-axis) versus vague prior with initial values from QNB estimates (x-axiy) for the 4 proteins (AMBP, B2M, C1S, and NRP2) whose model could not be obtained with random initial values and vague prior.


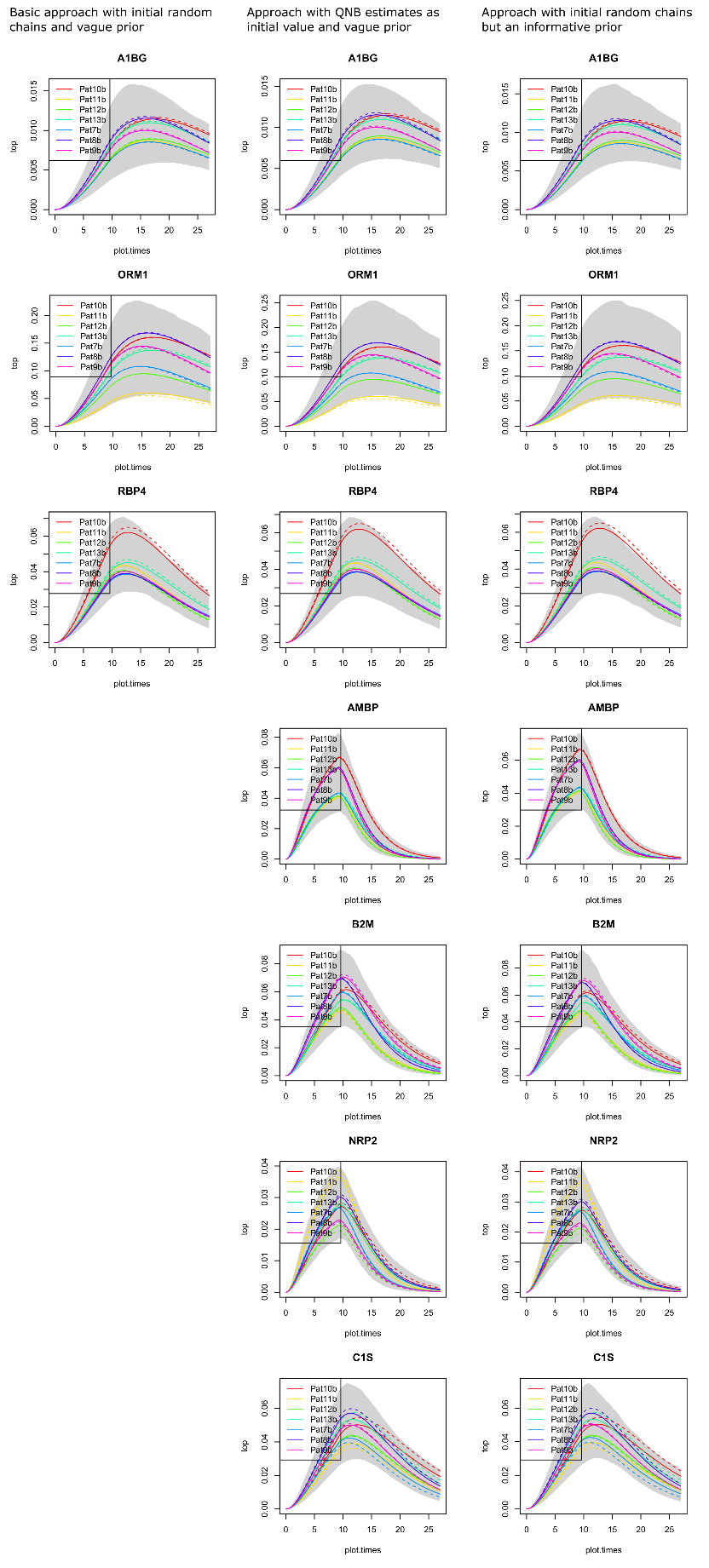


**Figure S7.** The population protein dynamics obtained with three approaches combining random or QNB given initial values for the Markov chains, or a vague *versus* a more informative (hyper-)prior on $\mu_{\theta}$. Three example plasma proteins whose population models were fit with all three approaches, followed by the four proteins whose models could not be obtained with the most basic and common approach. Gray areas represent 95% of the population posterior predictive distribution.


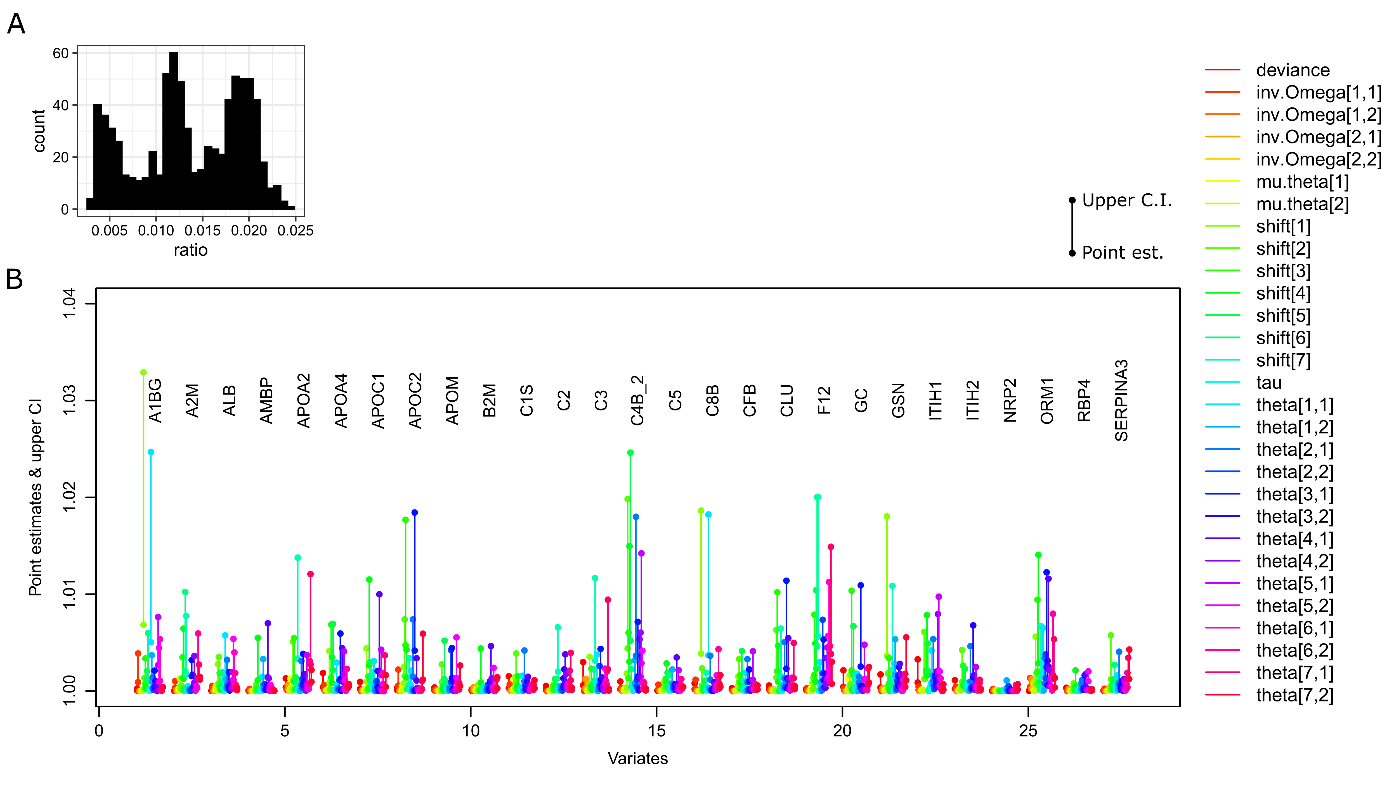


**Figure S8.** Population model convergence with plasma data. (**A**) Autocorrelation-corrected MCSE/estimate standard deviation distributions for all the model parameters pooled remained under 5% with 50,000 iteration after initial burn-in of 50,000 iterations as well. (**B**) Convergence according to Brooks and Gelman criterion (1998). Two Markov chains were run in parallel and the within- versus inter-chain variability ratio was computed. Point estimates along with the upper boundaries of 95% confidence intervals are plotted for all the model parameters and all the proteins. We see that all the point estimates and all the upper boundaries of CI95 remained below 1.05. The various model variables were named according to BUGS code, see Supplementary Methods below.


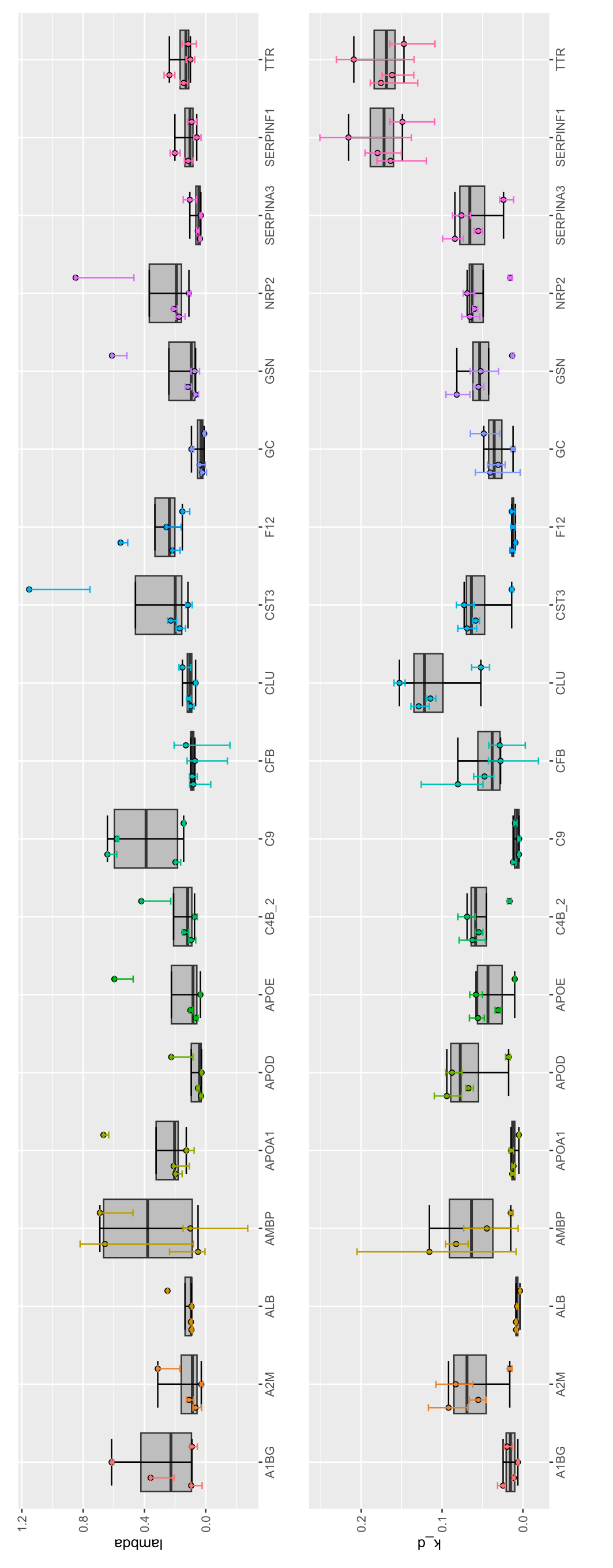


**Figure S9.** CSF individual model parameter variability.


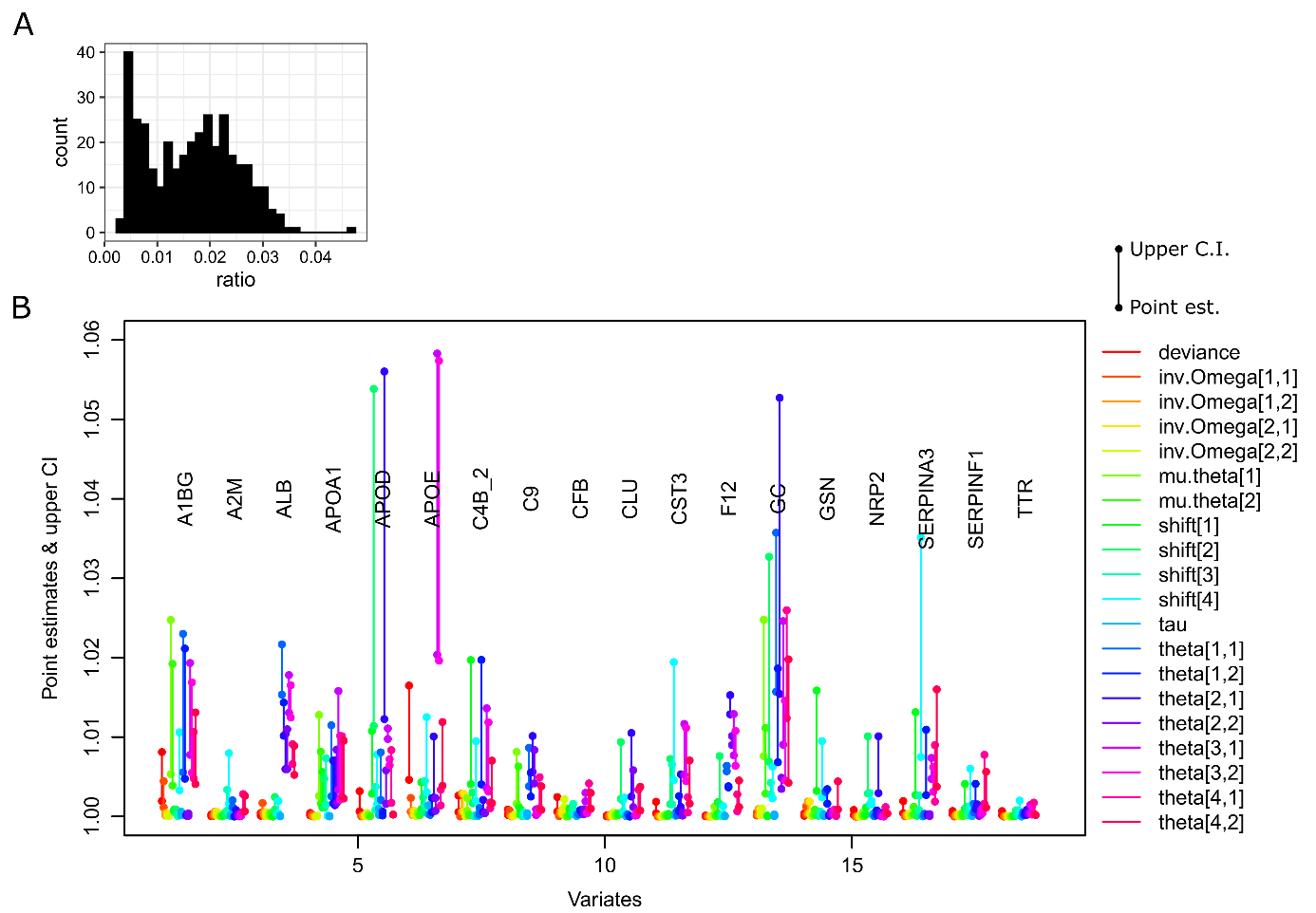


**Figure S10.** Population model convergence with CSF data. (**A**) Autocorrelation-corrected MCSE/estimate standard deviation distributions for all the model parameters pooled remained under 5% with 50,000 iteration after initial burn-in of 50,000 iterations as well. (**B**) Convergence according to Brooks and Gelman criterion (1998). Two Markov chains were run in parallel and the within- versus inter-chain variability ratio was computed. Point estimates along with the upper boundaries of 95% confidence intervals are plotted for all the model parameters and all the proteins. We see that all the point estimates and the majority of the upper boundaries of CI95 remained below 1.05. The few CI95 upper boundaries above 1.05 were below 1.06. The various model variables were named according to BUGS code, see Supplementary Methods below.

**SUPPLEMENTARY METHODS**

**Individual protein BUGS model**

# parameters: ngrid, ndim, init, times, origin, tol, llambda.mu,

# llambda.prec, lk_d.mu, lk_d.prec, shift.mu, shift.prec, weights,

# i_times

# ODE declaration

solution[1:ngrid,1:ndim] <- ode(init[1:ndim], times[1:ngrid],

D(C[1:ndim],t), origin,tol)

# ODE model priors

llambda ~ dnorm(llambda.mu, llambda.prec)

lambda <- exp(llambda)

lk_d ~ dnorm(lk_d.mu, lk_d.prec)

k_d <- exp(lk_d)

shift ~ dnorm(shift.mu, shift.prec)

# residuals and ODE definition

ilc <- step(9-t)

D(C[1], t) <- (lambda * ilc - C[1]) * k_d

D(C[2], t) <- k_d * (C[1] - C[2])

for (i in 1:n){

j[i] <- i_times[i]

mu[i] <- solution[j[i], 2] - shift

w.tau[i] <- tau * weights[i]

ratios[i] ~ dnorm(mu[i], w.tau[i])

}

# residuals prior

tau ~ dgamma(0.001,0.001)

**Likelihood function for adaptMCMC or mcmc R libraries**

# diff model ------------------------------

library(deSolve)

ilc <- function(t){

if (t <= 9)

1

else

0

}

model.2.1.j <- function(t,y,params){

Alpha <- y[1]

Beta <- y[2]

dAlpha <- (params[1]*ilc(t)-Alpha)*params[2]

dBeta <- params[2]*(Alpha-Beta)

list(c(dAlpha,dBeta))

}

# likelihood

lupost <- function(par,ratios,pp){

# parameters

llambda <- par[1]

lk_d <- par[2]

shift <- par[3]

sigma <- par[4]

if (sigma<=0)

return(list(log.density=-Inf))

# log-prior

lprior <- dnorm(llambda,pp$llambda.mu,llambda.sd,log=T) +

dnorm(lk_d,pp$lk_d.mu,lk_d.sd,log=T) +

dnorm(shift,shift.mu,shift.sd,log=T) +

dgamma(1/sigma**2,shape=0.001,rate=0.001,log=T) # gamma prior for the precision

# log-likelihood

times <- sort(unique(c(0,9,ratios$times)))

parameters <- exp(par[1:2])

ode.out <- radau(y=c(Alpha=0,Beta=0),times=times,func=model.2.1.j,

parms=parameters,events=list(time=9))

LL <- 0

for (i in 2:nrow(ode.out)){

good <- ratios$times==ode.out[i,1]

if (sum(good)>0)

LL <- LL+sum(dnorm(ode.out[i,3]-ratios$ratios[good]-shift,mean=0,sd=sigma,log=T))

}

list(log.density=LL+lprior)

} # lupost

**Population BUGS model for 7-patient plasma population**

# ODE system declaration

solution[1:ngrid,1:ndim] <- ode(init[1:ndim],times[1:ngrid],D(C[1:ndim],t),origin,tol)

# ODE parameters with their priors

for (i in 1:npat){

lambda[i] <- exp(theta[i,1])

k_d[i] <- exp(theta[i,2])

theta[i,1:2] ~ dmnorm(mu.theta[1:2],inv.Omega[1:2,1:2])

}

# hyper-priors of the ODE parameters

mu.theta[1:2] ~ dmnorm(mu[1:2],T[1:2,1:2])

inv.Omega[1:2,1:2] ~ dwish(R[1:2,1:2],2)

# injection of heavy leucine

ilc <- step(9-t)

# ODE definitions

D(C[1], t) <- (lambda[1] * ilc - C[1]) * k_d[1]

D(C[2], t) <- k_d[1] * (C[1] - C[2])

D(C[3], t) <- (lambda[2] * ilc - C[3]) * k_d[2]

D(C[4], t) <- k_d[2] * (C[3] - C[4])

D(C[5], t) <- (lambda[3] * ilc - C[5]) * k_d[3]

D(C[6], t) <- k_d[3] * (C[5] - C[6])

D(C[7], t) <- (lambda[4] * ilc - C[7]) * k_d[4]

D(C[8], t) <- k_d[4] * (C[7] - C[8])

D(C[9], t) <- (lambda[5] * ilc - C[9]) * k_d[5]

D(C[10], t) <- k_d[5] * (C[9] - C[10])

D(C[11], t) <- (lambda[6] * ilc - C[11]) * k_d[6]

D(C[12], t) <- k_d[6] * (C[11] - C[12])

D(C[13], t) <- (lambda[7] * ilc - C[13]) * k_d[7]

D(C[14], t) <- k_d[7] * (C[13] - C[14])

# residuals

for (k in 1:npat){

for (i in offset[k]:(offset[k+1]-1)){

j[i] <- i_times[i]

mu.resid[i] <- solution[j[i],2*k]-shift[k]

w.tau[i] <- tau*weights[i]

ratios[i] ~ dnorm(mu.resid[i],w.tau[i])

}

shift[k] ~ dnorm(shift.mu,shift.prec)

}

# residuals prior

tau ~ dgamma(0.001,0.001)
